# A System of Anatomy for the Use of Students of Medicine

**Published:** 1839-02-09

**Authors:** 


### BIBLIOGRAPHICAL NOTICE.

*A System of Anatomy for the Use of Students of Medicine.* By Caspar Wistar, M. D., late Professor of Anatomy in the University of Pennsylvania. *With Notes and Additions*, by W. E. Horner, M. D., Professor of Anatomy in the University of Pennsylvania. *Seventh Edition, entirely Remodeled, and Illustrated by numerous Engravings.* By J. Pancoast, M.D., Lecturer on Anatomy and Surgery, &c. &c. &c. In two Volumes. Philadelphia: 1839. 8vo. pp. 491, 560.

The "Anatomy" of the late Professor Wistar has long since assumed that station in medical literature, and in the estimation of the profession to which the talents and acquirements of its distinguished and revered author entitled it. Since his death, several editions have appeared under the superintendence of his friend and successor, Prof. Horner. The present, which is the seventh, has been prepared for publication by Dr. Pancoast, who retaining both the original context and the valuable notes of Prof. Horner, has remodeled the entire work, and added some recent important discoveries in this department of our science. We are, in general, pleased with the manner in which Dr. Pancoast has discharged his duties. He has displayed commendable industry in his researches, and much judgment in the selection and arrangement of his copious materials. His style is not always so neat or so clear as we could desire, nor are his descriptions invariably correct; as for example, when he states the insertion of the longitudinal muscular fibres of the rectum, first accurately defined by Professor Horner, to be into "the inner face of the mucous membrane," (vol. 2. p. 58,) instead of into its periphery. The chief contributions of the present editor have been made to the department of general anatomy, and, with few exceptions, they represent faithfully the actual condition of our knowledge in this branch. Two chapters on neurology have been added. We regret that the editor did not bestow more labour and time on their preparation. They have evidently been hastily compiled, and are too brief to do full justice to this important and interesting subject.

Although the type and paper are of excellent quality, adequate attention has not been paid to the correction of the press, and the sense is frequently perverted or obscured by typographical errors. At page 492, we have the same paragraph twice in half a page. The illustrations are very well executed.

In the chapter on the "General Anatomy of the Mucous Membranes of the Alimentary Canal," the editor has inadvertently given credit to Drs. Bœhm and Boyd, for discoveries in its intimate structure, which were promulgated here long before the labours of either of these gentlemen reached this country. Professor Horner, of the University of Pennsylvania, while pursuing his researches into the pathology of Asiatic cholera, during the autumn of 1834, was induced to examine, minutely, the organization of the gastro-enteric mucous membrane, and more especially its glandular apparatus. These investigations led him to believe that our knowledge on this subject had been hitherto exceedingly imperfect and crude, and, in many respects, erroneous. The result of his own repeated and careful microscopic observations were as follows :

The mucous membrane of the stomach and intestines, he found to consist principally of a cribriform venous intertexture, which he proposed to call the *superficial venous layer*. Beneath this there is an arterial and venous arborescence, the venous ramuscles of this deep-seated layer, receiving the blood of the superficial one, their origin being bent vertically towards the mucous surface. The character of the *superficial venous layer* varies with its location, and its appearance is very unlike a common vascular anastomosis, and in the colon resembles a metallic plate, numerously pierced with circular holes. These foramina are in fact the mucous follicles, which penetrate as deep as the arterial-venous layer. By inverting the intestine, and then inflating it, the peritoneal coat being removed, the existence of an epidermis throughout the stomach and intestines will be incontestibly proved. On the erection of the villi by injection, those in the jejunum are found to resemble the cerebral convolutions. In the ileum they become mere conical projections, and near the ileo-colic valve they disappear entirely. No villi exist in the stomach or colon. At a moderate computation, the entire number of follicles in the alimentary canal is upwards of forty-six millions nine hundred thousand. In the stomach, the largest of these are one-ninety-eighth of an inch in diameter, and the smallest one four hundred and ninetieth. In the colon, the largest is about one two hundred and forty-fifth of an inch in diameter, and the smallest about one four hundred and ninetieth. From the location and structure of these follicles, Dr. H. is inclined to adopt the opinion of their absorbent faculties. He failed to detect any orifices to Peyer's glands, they appearing merely as small lenticular excavations, disturbing the natural arrangement of the villi. How closely these views coincide with those of Drs. Bœhm and Boyd, in numerous particulars, we need not add.

In the first number of the British and Foreign Medical Review, published in London, January, 1836, Dr. Horner's paper, "On the Anatomical Character of Cholera," which appeared in the American Journal of the Medical Sciences, for May, 1835, and which contained the novel anatomical views just detailed, was partially copied, with the subjoined editorial comment. "To do full justice to Dr. Horner's opinions, *it would be necessary to detail his views of the anatomical structure of the mucous membranes.* But this we cannot do in this place." (Vol. I., pp. 236–7.) Now on the same page, is an article headed, "On the Intimate Structure of the Intestinal Glands, by Dr. Bœhm," from Hecker's Annals. This was the first intimation the British public had of the researches of the German anatomist, and they were, at this time only, briefly adverted to. In a subsequent number, there was a full analysis of Dr. Bœhm's thesis, but no mention was made of the similar investigations of the American Professor, although his communications must have reached England some time previously to that of Dr. Bœhm's. Dr. Sprott Boyd's essay was not published in Edinburgh until the latter part of 1836.

